# Corporate Social Responsibility in Liquid Times: The Case of Romania

**DOI:** 10.1007/s10551-021-04926-w

**Published:** 2021-08-30

**Authors:** Georgiana Grigore, Mike Molesworth, Andreea Vontea, Abdullah Hasan Basnawi, Ogeday Celep, Sylvian Patrick Jesudoss

**Affiliations:** 1 School of Business, University of Leicester, Brookfield Campus, Leicester, LE2 1RQ UK; 2 Henley Business School, University of Reading, Greenlands, Henley-on-Thames, RG9 3AU UK; 3 Bucharest University of Economic Studies, Piața, Romană 6, 010374 Bucharest, Romania; 4 Henely Business School, University of Reading, Whiteknights, Reading, RG6 6UD UK

**Keywords:** Corporate social responsibility, Zygmunt Bauman, Adiaphora, Liquid modernity, Romania

## Abstract

Existing scholarly work on corporate social responsibility (CSR) frequently emphasizes either normative/ethical claims about social progress or instrumental/strategic claims about corporate effectiveness, yet less often acknowledges the moral conditions of those undertaking CSR within a specific cultural context. In this paper, we draw attention to the social conditions in which CSR takes place and the related ethics of the subjects that must enact it. Our approach is to document the lived experiences of practitioners in Romania, a post-communist society. Drawing from fifty-three depth interviews with both corporate responsibility practitioners, and managers in non-profit organizations who together work on CSR projects, we describe their experiences of the social and organizational environment, the CSR practices that are undertaken in this context, and the intended and unintended consequences of such work. Using Bauman’s theorization of ethics, including adiaphora and moral distancing, and Borţun’s interpretation of Romanianness, we then theorize liquid CSR as an ambivalence between adiaphoric practice (instrumental morality, careerism and self-interest) and the moral impulse to do good, resulting in both intended (short-term promotion and competitive victimhood) and unintended consequences (a potential for corruption and collateral beneficiaries).

## Introduction

Since the fall of communism, Central and Eastern European societies have been developing political and economic approaches that integrate Western norms, in part to achieve newly imposed EU standards (Soulsby & Clark, 2007; Soulsby et al., 2017; Stoian & Zaharia, 2012). Policies and practices have been imported from developed European nations (Soulsby & Clark, 2007), including the responsible business practices of multinationals, especially through local subsidiaries (Stoian & Zaharia, 2012) and consistent with the adoption of US-style ‘explicit’ CSR as suggested by Matten and Moon (2008). Yet practices from the communist era and before still remain in the collective memory (Borţun, 2011; Tileagă, 2018) and, as Bauman and Donskis (2013, 2016) have noted, the ethical basis of Western institutions has itself eroded, raising doubts about how adopting their approaches might lead to corporate responsibility in former communist societies. The result is CSR that incorporates recent Western ideas, but that also reflects a morality derived from local cultures, politics, and social institutions. Central and Eastern European CSR may not, therefore, be fully understood through established theories, and indeed an examination of these separate cultural contexts may produce new theorizations (see Arnould et al., 2006). This study therefore addresses the need for CSR research in developing countries (Ahmad et al., 2012; Grigore et al., 2020; Jamali et al., 2017; Kim & Kim, 2010), including Eastern European transition economies (Crotty, 2016; Stoian & Zaharia, 2012). Romania—our focus—provides a unique context that reveals aspects of CSR that are less easily observed in developed Western economies where the majority of CSR research has been undertaken.

We aim to: (1) identify how the cultural context in Romania shapes CSR practices ‘on the ground’; (2) understand the reported consequences (both intended and unintended) of such practices, and; (3) theorize Romanian corporate responsibility in a way that allows further critical reflection on the project of CSR itself.

We draw from fifty-three interviews with people working in what Tams and Marshall (2011) call ‘responsible careers’, where individuals tackle ethical, environmental or societal issues in their work. These included corporate managers, and managers of non-profit organizations who work on CSR projects in collaboration with corporations (the dominant structure of CSR in Romania). Recognizing Borţun’s (2011) interpretation of Romania’s culture and using Bauman and Donskis’s (2013, 2016) critique of the morality of contemporary neo-liberal societies, including symptoms of moral distancing and adiaphora, we contribute to knowledge by theorizing *liquid CSR,* based on careerism and self-interest, and a stifled moral impulse to do good. This results in both intended consequences (short-term promotional opportunities based on competitive victimhood) and unintended consequences (a potential for corruption, and for *collateral beneficiaries*). This critical perspective argues that when the morality of CSR in Romania is understood from the ground, there is reason to doubt both instrumental/strategic and normative/ethical claims for CSR. In Romania, CSR practices are a result of the legacy of communism—that forestalled an already limited project of modernization—meeting the dissolution of ‘top down’ modernism in less secure and more liquid times, the result is CSR that is precarious, promotional, bureaucratic, subject to arbitrary change, and outside of moral concerns.

We start with a review of CSR research, noting instrumental/strategic claims about its effectiveness, normative/ethical claims about its morality, and recognition that the purposes and practices of CSR vary by cultural context. We then introduce Bauman’s ethics as a framework that can help us to understand the moral conditions of CSR practice. We then explain the Romanian context and our methods. Next, we provide stories from the participants, organizational processes, the experiences of CSR projects and the outcomes of CSR. Finally, through our interpretations, we offer a theorization of *liquid CSR* in Romania.

## CSR, Morality and Culture

Much research seeks to justify the business case for CSR and so present it as a ‘win–win’ approach where both society and business benefit from developing strategic, responsible practice (Porter & Kramer, 2006). The business case for CSR has expanded through such instrumental claims (Barnett, 2016), including how CSR creates competitive advantage (Porter & Kramer, 2011), increases financial performance (Luo & Bhattacharya, 2006), and enhances brand awareness and employee retention (Bhattacharya et al., 2009), none of which would usually be related to ethical practice.

Instrumentalism therefore distances CSR from the discourse of moral responsibility such that CSR theory has limited foundation in ethics (Roberts, 2003). The focus of research is also on the business, rather than the moral condition of CSR practitioners (see Pedersen, 2010). Yet questions about ethics are hard to evade in a discourse that persistently talks of ‘doing good’ and, as Baden and Harwood (2013) point out, once CSR loses any ethical foundation, it can only serve to distract from historically proven solutions to social and environmental impacts, such as regulation. A normative/ethical approach to understanding CSR has therefore also emerged.

For example, Van de Ven (2008) has explored what a virtuous company is when it comes to CSR communication, suggesting a tendency to *consequentialism* in CSR promotions (where ‘everyone wins’), that leads to arbitrary selection of causes based on communication strategy. Van de Ven (2008, p. 342) contrasts this with a *virtue-based* approach where the motive for CSR—and not just the outcome—is considered, concluding that: “if the interest of the firm is the sole motivation to engage in CSR initiatives (a narrow-minded profit orientation) one could rightly object from a virtue ethical perspective that this diminishes the moral value of the initiative”. Frederiksen (2010, p. 357) also considers the moral philosophy underpinning CSR, noting that: “we need knowledge about the moral foundation of CSR if we want to discuss whether that moral foundation seems reasonable”. Based on managers’ responses to imagined cases, the author found that *common-sense morality* rules, including financial obligations towards shareholders, but also physical and social proximity principles, i.e. that business prefer to support local communities in areas where the organization has existing expertise or involvement. Frederiksen (2010) further notes that the morals suggested by companies’ policies are inconsistent with the moral foundations of managers, highlighting a potential gap between what organizations communicate and what managers themselves experience.

Köllen (2016) picks up the issue of underlying morality by drawing from Schopenhauer to explore egoism, compassion and malice as motivators for action, noting a tendency for CSR to be used as a criterion for moral evaluations (along with diversity). Hence an organization is seen as moral if it undertakes CSR, without reference to moral philosophy. This has the effect of taking morality away from the individual and placing it in the ‘virtuous organization that does CSR’.

For Baden and Harwood (2013), corporations interpret CSR in their own interests, creating a suspicion that it is merely a legitimizing myth (Devinney, 2009), and part of an established neo-liberal script (Schneider, 2014), with little reference to the norms of citizenly behaviour (Garriga & Melé, 2004). These are therefore also persistent concerns that CSR practice does little to address the negative consequences of capitalism (Bradshaw & Zwick, 2016). Such critical CSR research highlights a potential gap between both normative and strategic claims about CSR and its actual outcome.

Work on strategic/instrumental CSR, ethical/normative CSR, and also critique of the CSR project, has resulted resulting in a dizzying 157 different definitions (Blowfield & Murray, 2008). Not surprisingly, Walters and Anagnostopoulos (2012) describe CSR as a “tortured concept”, confused by contradictory claims. One ‘solution’ to such diversity is to recognize CSR as a concept that manifests in a wide range of contexts and cultures as a result of both globalization and the culturally situated moral conditions of those who undertake it. For example, the cultural context of morality is implied in Matten and Moon’s (2006) explanation that the US adopted explicit CSR because of limited state co-ordination of labour, health and education that provided space for CSR initiatives, whereas the EU only adopted similar explicit CSR as it marketized social welfare, with changes in corporate finance (capital markets) also resulting in explicit CSR that meets the criteria of investors. The EU has recently encouraged explicit CSR at the same time as restricting policies that produce implicit CSR in accession countries by insisting on fiscal prudence. By considering institutions, Matten and Moon (2006) reveal how CSR is shaped by public policy in the environment, health and education, and the local role of business in society. Key here is that CSR is enacted differently in different countries, but is also subject to a more general move to neo-liberal policies globally.

The recognition that CSR and its moral underpinning vary by nation is also seen in studies that expand the North American empirical focus, often drawing from Hofstede’s cultural dimensions. For example, Stajkovic and Luthans (1997) note the need to ground philosophical/theoretical work in actual business practice, creating a model based on Bandura’s social learning theory, and informed by Hofstede, that highlights institutional, organizational and personal factors in the morality of business in different cultures. Sims and Gegez (2004) consider prior research on US, Israel, Australia and South Africa and compare this with Turkey using the Attitudes Towards Business Ethics Questionnaire (ATBEQ), a corruption index, and Hofstede’s dimensions, applied to a sample of business students. By comparing ‘cultures’ they theorize how specific cultures are more or less willing to engage in unethical business. Sanyal (2005) deals specifically with bribery as an indication of business ethics, noting that it is more likely in countries with low per capita income, and is determined by cultural factors such as high power distance and high masculinity. They further suggest that countries undergoing political, economic, and social changes—as witnessed in Eastern Europe and the former Soviet Union—are especially vulnerable to corruption. Kim and Kim (2009) also draw from Hofstede in a Korean study, but recognize limitations of Hofstede in explaining practitioners’ perceptions of CSR. Alternatively, Freeman and Hasnaoui (2011) seek a consensus definition of CSR by examining similarities across four Western countries, aiming to create a universal framework. Again, using Hofstede, they present an ‘evolution’ metaphor that suggests internationalization harmonizes cultures in the UK, the US, Canada and France when it comes to business responsibilities. As a final example of Hofstede-based work, Dam and Scholtens (2013) apply Hofstede’s cultural dimensions to a range of country contexts to understand how culture affects ethical policies at organizations, finding that individualism and uncertainty avoidance are positively associated with the existence of ethical policies, whereas masculinity and power distance are negatively related.

Despite the enduring popularity of Hofstede’s cultural dimensions in management research, there are established criticisms of the approach. Baskerville (2003) provides a well-cited overview of these. Hofstede’s theory equates nation with culture, ‘averaging out’ cultural values within a single nation. Indexing also quantifies what sociologists and especially anthropologies recognize as a domain requiring rich description above simple reductionism (outside of management studies Hofstede is seldom used). The result is that Hofstede-based approaches actually minimize differences between cultures (e.g. individualism becomes the same in all countries where it is measured) and then present ‘culture’ as a variable to measure, say, approaches to ethics, rather than a context in which new theory can be generated. As Baskerville (2003) notes, this neglects *understanding-from-within a culture* in favour of *measuring-from-without*. In any case, Baskerville also highlights that international comparisons are better done through socio-economic measures and not reified dimensions of culture. The conclusion is that other anthropological or sociological approaches may provide better explanations of cultural differences in business activity.

Hofstede-based comparisons tell us little of the *lived experience* of any culture, or the form that ethical practice takes, by reproducing the cultural dimensions rather than providing alternative theorizations of the area under investigation. Approaches that reject Hofstede are seen in CSR research, however. For example, Wang and Juslin (2009) consider how cultural context produces local versions of practice best understood through related philosophy, in this case ‘Confucian interpersonal harmony’ in China. They note the relatively recent development of local CSR based on the needs of MNC, including concerns in the West about Chinese manufacturing practices. However, they specifically highlight the limitations of applying Western CSR concepts, and a tension between a desire for a single (US-based) definition of CSR, and the maintenance of cultural uniqueness in theory. Yin and Zhang (2012) also recognize the bias towards Western theory and contexts in CSR research, arguing that practices themselves remain ‘local’, in this case again informed by specific Chinese philosophies and attitudes to both business and morality.

By considering Confucianism, Wang and Juslin (2009) conclude that corporate responsibility existed in China long before Western MNC imported of the idea. Boardman and Kato (2003) take a similar approach by deploying a traditional Japanese concept—Kyosei—to understand CSR, again evoking ‘ancient’ philosophy as a metaphor for contemporary practice. A risk here, however, is of dividing the world into dualisms of West versus East (or versus former communist), or developed versus developing (or versus transition), conflating broad geographies and global development movements with cultural categories. Nevertheless such research does show how original CSR theories can be generated by examining practices within a specific culture. This is further illustrated by Crotty’s (2016) study of CSR in Russia. Crotty notes that Russian CSR diverges significantly from that described in other contexts (including other former communist countries), because it relates more closely to compliance with legal issues in a society where legislation that ensures responsibility is precarious. Hence Russian CSR is not necessarily ‘beyond compliance’ as it might be in the West, and is not understood as only a Western import, but also recognized as an aspect of central planning, i.e. as embedded in cultural, legal, and institutional frameworks, including the recent complexity of a ‘lawless’ transition period, and the possibility of ‘coerced’ CSR projects forced on businesses by the state.

This presents an alternative approach to researching cultural context that is not about normative comparisons, but about broadening an understanding of a diverse practice. Hence, we are not interested in how like or unlike ‘the West’ (or even ‘the East’) Romania is, but what the Romanian context reveals about the possibilities of the CSR project. Arnould et al. (2006) highlight how cultural contexts generate theory by evoking specific emotions and senses, stimulating discovery (a sense that something different is happening), and exciting theoretical comparisons (by foregrounding or backgrounding particular arguments). In our case, the enthusiastic but chaotic descriptions of participants background both strategic/instrumental and normative/ethical CSR issues, inviting new theorisations. As Arnould et al. (2006) also highlight, ‘extreme’ contexts are especially useful for suggesting new metaphors and theories. As we have seen, often these are derived from ancient philosophies that risk a nostalgic re-creation of culture in light of new business developments such as CSR, or foreground an older structuring of morality in a culture that under-represents recent development. We therefore suggest that contemporary commentary on modernity—Bauman’s ethics in Liquid Modernity—provides a more suitable theoretical/metaphorical lens with which to interpret Romanian CSR.

## Zygmunt Bauman’s Ethics

Zygmunt Bauman’s theories relating to ethics of responsibility in contemporary societies have been articulated over several decades, starting with questions about the role of ordinary individuals in the Holocaust (Bauman, 1989), later covering aspects of business and consumption (Bauman, 2001, 2007) and most recently, discussed with Leonidas Donskis in *Moral Blindness* (Bauman & Donskis, 2013) and *Liquid Evil* (Bauman & Donskis, 2016). He has also been an important critical reference in business ethics and CSR research (Jensen, 2010, 2014; Roberts, 2003; Ten Bos, 1997). In presenting Bauman’s work, we note, as do Roberts (2003) and Jensen (2010, 2014), an apparent gap between the claims made for CSR as ethical business practice and the moral conditions under which businesses operate.

Bauman’s (2000, 2001) key works describe *liquid modernity* through observations that progress has moved from ‘top down’ to ‘bottom up’. Previously, citizens saw solid institutions (such as governments) as accountable for social progress, but now progress has become uncertain and individualized (Bauman, 2000, 2001). Grand narratives of modernism, captured through rising living standards, economic growth and social improvements, have given way to individuals who are alone and in it for themselves, competing in an environment where problems are created by markets as much as they are solved (Bauman, 2007; Jensen, 2010; Roberts, 2003).

We now capture several key themes in Bauman’s discursive work that represent a tendency for moral distancing that can lead to what he refers to as a modern version of *adiaphora*, the areas of life placed outside ethical judgement. Bauman’s ideas allow for re-evaluation of CSR, and such observations are further problematized in the Romanian context, which Borţun (2011) notes never developed a fully modernist belief in its institutions (see below).

### Moral Distancing

Bauman (2001) observes that it has become a duty of individuals to compete for their own progress in life. The implications are widespread but are especially important in employment with a competitive workforce using each job as a stepping stone to the next promotion, complying with bureaucratic demands (contractual loyalty, as Jensen, 2010, sees it), while showing no responsibility other than to their career, and with each move improving salary, status and work contacts. The result is an episodic, or projectized life, with no purpose other than to achieve for oneself (Bauman & Donskis, 2013, 2016).

The individual’s moral impulse is subsequently replaced by adherence to the management rules that define success (Bauman & Donskis, 2013). Work becomes remote from its consequences, with actions that affect people hundreds or thousands of miles away taking place in offices and on computer screens (Bauman & Donskis, 2013, 2016; Jensen, 2014). Jensen (2010) also observes how a specialization of labour splits the person so that individuals are incapable of ethical action, only of following the organizational rules for their specific role. Everything becomes somebody else’s problem, or fault (Bauman & Donskis, 2016).

Responsibility to another is therefore a key casualty of societies where relationships become morally distant and “pure”, meaning “no strings attached” and “no unconditional obligations” (Bauman & Donskis, 2013:14), and if there is no longer individual responsibility for others, we might also question how there could be corporate responsibility. As Jensen (2010, 2014) points out, morality is always a matter of the individual, and moral philosophers like Singer (1995) have previously argued convincingly of the difficulty in actions being both instrumental (for some other purpose) and ethical, defining doing good as a spontaneous act of care for the other. Yet Bauman and Jensen argue that layers of instrumentalism in contemporary organizational work—the reduction of the individual to a role or traits, the desire for career progression, the performance review, the key performance indicator, and the profit goal—limit the moral impulse that comes from the direct experience of another. Drawing from Levinas, Bauman (2001) describes a sociology of ‘being with’, but no longer ‘being for’ the other. Relationships are temporary, based on a desire for individual success, and dropped when not useful. Morality is towards those that matter, with an instrumental focus on the self. For Roberts (2003), this ‘narcissistic morality’ potentially defines CSR as an instrumental response to the organizational need to be seen as good.

Bauman and Donskis (2013) further highlight that the problems of liquid modernity are especially apparent in post-communist societies, where the pace of (neo-liberal) change has been so fast, that there has been no time to locate change within history. Under liquid modernity, structures become temporary, ever changing (and therefore angst-ridden and insecure), with the result that everyone is in it for themselves right now and unable to consider the consequences of their actions.

### Adiaphora

For Bauman (2011), ethics is evaded by corporations, as any harm done to people through capitalism is dismissed as ‘collateral casualties’, unintended and unavoidable, and hence nobody’s fault (see Jensen, 2010, 2014). As the individual becomes distant, faceless and reduced to traits (a consumer need, an employee role, a stakeholder concern), they are no longer a subject for moral concern (Bauman & Donskis, 2013). This results in growing *adiaphora*—that which is outside moral concern, including adiaphoric companies and organizational members (Jensen, 2010, 2014). Bauman and Donskis (2013) note the original Stoic form of adiaphora as “things which are in reality inessential and unimportant” (p. 27), “cast outside the universe of religious and moral obligations”, and so “exempted from evaluation” (p. 51), but define *contemporary adiaphorization* as:

“Stratagems of placing, intentionally or by default, certain acts and/or omitted acts regarding certain categories of humans outside the moral-immoral axis – that is outside ‘the universe of moral obligations’ and outside the realm of phenomena subject to moral evaluation; stratagems to declare such acts or inaction, explicitly or implicitly, ‘morally neutral’ and prevent the choices between them from being subject to ethical judgement.” (Bauman and Donskis 2013, p. 40)

For example, markets favour an apparently morally neutral discourse of competitiveness. The language is of costs and benefits, and not ethics (Jensen, 2010). Common management terms capture this obscuration. For example, ‘cutting costs’ conceals potential suffering from unemployment or from exploitation of sweatshop conditions, which become mere unintended consequences of the process of saving money.

Bauman and Donskis (2016) reflect on the implications of these arrangements, noting that actions are defended on the basis that ‘there is no alternative’ and resulting in a loss of imagination about the future, and a reduction of responsibility. Individuals also experience a societal crisis as an onlooker of a ‘carnival’ that is dramatic and short-term. Suffering becomes competitive and overused, and compassion fatigue demands that any subsequent crisis needs to be more spectacular to register in our collective memories (Bauman & Donskis, 2016). Crises end up tragically competing with each other.

Despite an emphasis on such spectacle, Bauman’s contemporary definition of adiaphora represents a moral indifference to what is happening in the world. CSR is presented as an antidote to this, yet reveals tensions within corporations that desire to remain distant from moral scrutiny, while promoting their responsibility, hence as Jamali et al. (2017) observe, CSR is ‘decoupled’ from organizational activity, which is no longer the focus of moral evaluations and so can carry on as usual. We are invited to reward corporations that forgo even the smallest profit for the benefit of those outside the organization (Rhodes, 2016; Sen & Bhattacharya, 2001). Yet in this logic, the broader critique articulated by Bauman easily gets lost. Corporations remain Friedman’s (1962) amoral, financial utility-seeking organizations, with instrumental acts of good confirming an absence of morality in liquid modernity rather than addressing its problems.

As Romania imports CSR as Western concept, it also imports the morality of CSR into ‘local’ ethical standards and norms (Matten & Moon, 2008). As Bauman (1989) notes, humans are born morally ambivalent; it is their socio-historic *and* institutional existence that produces their moral responses to others. The task then, is to unpack how CSR as a situated cultural practice is undertaken, and to what moral end. Bauman’s description of morally blind, fragmented and agonistic modernism may seem overly bleak, and more rhetorical than empirical, yet it offers a useful frame for reflection on the stories about CSR provided by our participants, and more broadly on the morality of CSR itself.

## The Romanian Social and Cultural Context

Much commentary about Romanian culture focuses on the legacy of communism. For example, Djuvara (2014) notes Ceauşescu’s ambitious modernization plans, but recognizes that these were for self-aggrandizement more than economic growth. Djuvara’s (2014) interpretation of Ceauşescu’s regime is that “lying became an instrument of government, [and] stealing—not only from the state, but from one’s own neighbours—appeared legitimate.” For Djuvara (2014), Romania emerged from communism with a tarnished morality. Alistair et al. (2017a) suggest that under communism, bribery was a common way to overcome shortages and deficiencies.

However, Boia’s (2013) history of Romanian consciousness notes a preference for authoritarianism that pre-dates communism and still facilitates nationalism in politics (and indeed such sentiment was one reason why Romania remained more autonomous that other Eastern Block countries). Boia (2013) also reminds us that although Romania voted to join the EU, the electorate were far from unified with some rallying to government calls such as: “We won’t sell our country!” and welcoming attempts to sign a treaty with the Soviet Union instead. This is more than nostalgia for communism. Ideas and values from communism mixed with older nationalist sentiments, but distinct between them, were not reflected on in public discourse. Tileagă (2018) observes the considerable effort that has gone into providing an officially ‘authorized’ collective memory of communism that might create a version of the past that can be accepted in a way that that allows a new democratic sentiment. Yet this process remains contested.

Morăraşu and Drugă (2016) also highlight changes in social classes following the fall of communism that produced “local barons”, politicians who use their position to accumulate ostentatious personal wealth. This created an environment which “promotes non-values: lying, flattering and viperish characters.” They further illustrate this as an enduring quality of Romanian public life, for example captured by Mihai Eminescu—Romania’s most influential poet—in *Scrisoarea III* (1881), where politicians are categorized as bedlamites and dastards. The Romanian newly rich are seen as just the latest iteration of this problematic group and ordinary Romanians remain suspicious of democratic political power, because corruption has so long been normalized in public life.

Borţun (2011) therefore rejects communism as a single cultural frame, arguing that there has been too much emphasis on communism in analysis of Romanianness, making it something of a scapegoat for ongoing problems, and resulting in too little critical attention to the national character. One result is a failure to recognize a tendency for Romanians to identify with *anything* that seems to characterize the country, including problematic aspects of society that are embraced as “the way we are” and that ensure that change is superficial. Borţun uses the metaphor of creating a pretty façade as a Romanian tendency.

For Borţun (2011), the brutality of Romanian communism did not result from Soviet influence, but from Romania’s previous failure to modernize. He again observes a façade of democratic citizenship that hides resistance to political progress. From this, he argues that it is problematic to assume that Romania can somehow catch-up with other EU countries because it is so unlike the countries it is trying to emulate. Romania understands modernism as the material development of infrastructure, but Borţun (2011) argues that modernism must also include social values and related political and legal systems; the changes in thinking that modernism produced in Western European countries. Romania remains locked in a feudal-like system—a pre-modern society—with local power bases maintaining their wealth and influence and a ‘peasant mentality’ focussed on ‘looking out for themselves’ (Borţun, 2011). This is therefore not the modern Western form of individualism, but a more closed and inward looking one, hence the tendency towards nationalism that has been appropriated by political forces to perpetuate local power, with an accompanying normalization of corruption justified as an indelible aspect of the national culture (Borţun, 2011). In short, Romania only pretended to modernize and uses the communist era as an excuse for failed progress that is actually part of an older and unchallenged national character.

Conditional arrangements following EU membership in 2007 have insisted on measures to improve political and legal systems, and to prevent corruption (Alistair et al., 2017a). This Cooperation and Verification Mechanism remains, highlighting the extent of the problem. Alistair et al. (2017a), further illustrate this with reference to Romania’s place in the Corruption Perception Index. Although Romania has moved from 87 (out of 146 countries) in 2004 to 57 (out of 176 countries) in 2016, in 2015, 29% of Romanians still admitted they paid a bribe in 2014 (Global Corruption Barometer 2016, cited in Alistair et al., 2017b). There are also persistent public demonstrations against corruption. For example, Rogalski (2017) notes how the 2015 fatal fire at the Colectiv nightclub in Bucharest (attributed to corruption in health and safely enforcement) energized a public outcry. Yet one subsequent political response was to prevent political corruption resulting in jail, and Rogalski (2017) states that successive governments have lacked the will to deal with the issue, resulting in calls for civil society to hold governments to account. Borțun and Cheregi (2017) also highlight that tensions have resulted in popular protests, where young Romanians demand “a country like the ones abroad” (largely free from corruption). The political and social divide in the resulting protests and then also against the president (accused of wanting to split the country), are seen as evidence of a failure to integrate the old (ethnically-based and embedded in sentiments of authoritarianism) and the new Romanians (politically and socially progressive and looking towards the EU for sources of progress). Romanian politicians are accused of claiming to represent the later, while persisting with the former.

Borţun (2015) has also applied his understanding of Romanianness to CSR, observing that managers naturally use CSR to gain reputation, especially where corporate interests may be inconsistent with new public sentiments around health or the environment. His analysis is that CSR in Romania tends to neglect meaningful engagement with social issues and instead focuses on an exaggerated version of instrumental CSR, where the claims made by corporations about their CSR activity are inconsistent with their actions. He supports such claims with observations that there is little external auditing of activity, and that much CSR operates out of marketing departments. He further notes that, “In a society in which, by way of tradition, neither the Church, nor the State have excelled in carrying out social responsibility activities, it would be worth learning to what extent CSR may become a source of social change…”.

## Research Design and Methodology

We aim to: (1) identify how the cultural context in Romania shapes CSR practices ‘on the ground’; (2) understand the reported consequences (both intended and unintended) of such practices, and; (3) theorize Romanian corporate responsibility in a way that allows further critical reflection on the project of CSR itself.

Although various methodologies have been deployed to construct knowledge of CSR, including surveys (Pedersen, 2010), experiments (Bhattacharya et al., 2009) and case studies (Rhodes, 2016), interpretivist approaches remain more limited (Khan & Lund-Thomsen, 2011). In focussing on the lived experiences of practitioners, our study responds to the need for practitioner-focussed CSR research (Pedersen, 2010) and for methodological pluralism (Davis et al., 2013), especially more interpretivist studies (Bass & Milosevic, 2016; Taneja et al., 2011).

In Romania, CSR activity is assembled through partnerships between corporations and NGOs, consistent with what Laasonen et al. (2012) describe as ‘dyadic partnerships’, and with the NGO-business relationships described by Maier et al. (2016). As Campbell (2007) suggests, in CSR, commercial, not-for-profit, governmental and inter-governmental actors come together to do good. One result is that Romanian NGOs have become entrepreneurial in their search for grants and funds, including by working with and like businesses (see Aßländer & Curbach, 2015; Maier et al., 2016). Indeed, most Romanian CSR projects are dependent on NGOs, and their number increased dramatically when multinationals introduced CSR to Romania. In 2016, the Romanian National Institute of Statistics identified 45,000 NGOs.

As a result, we interviewed more participants from the non-profit sector than from corporations, having sought individuals who describe *themselves* as working on CSR in their role, or what Tams and Marshall (2011) conceptualize as “responsible careers”. In Romania, people working in NGOs actually implement most of the CSR activity. We combine the narratives of both NGO and corporate managers as they talk about a range of projects, to provide accounts of how Romanian CSR practice happens ‘on the ground’.

Between November 2013 and January 2016, we conducted 53 depth interviews held in the offices of participants or in coffee shops in Bucharest, generating approximately 58 h of data. Most multinationals have their headquarters and related CSR departments in Bucharest, but they implement CSR projects across the country. Five participants were interviewed twice (in 2013, and then in late 2015, or early 2016) following up on accounts provided about changes in their position, or job, or ongoing projects. Position titles included: corporate responsibility manager, corporate communications manager, community affairs manager, president, director, executive director, fundraising manager, community strategy or relations manager, programme coordinator. Industry sectors included: automotive, FMCG, retail, construction, banking, tobacco, technology, and non-profit organizations that support community, health, environmental, minority groups, human or animal rights issues (see Table 1).

**Table 1:** Depth interviews conducted between November 2013 and January 2016

No.	Pseudonym	Position	Organization	Sector
1	Beatrice	Community Affairs Manager	Bank	For-profit
2	John	Corporate Communications Manager	FMCG Corporation	For-profit
3	Tony	Corporate Responsibility Manager	Cosmetics Corporation	For-profit
4	Diana	Communications Manager	Cosmetics Corporation	For-profit
5	Valeria	Communications Manager	Consulting Company	For-profit
6	Florin	Programme Coordinator	Minority Groups NGO	Non-profit
7	Georgina	Community Strategy Manager	Health and Community NGO	Non-profit
8	Mugur	Project Manager	Human Rights NGO	Non-profit
9	Sarah	President	Environmental NGO	Non-profit
10	Laura	Corporate Relations Managers	Global Environmental NGO	Non-profit
11	Dumitru	President	Environmental NGO	Non-profit
12	Roxana	President	Minority Groups NGO	Non-profit
13	Iulia	Corporate Affairs Manager	Tobacco Corporation	For-profit
14	Sabina	Community Relations Manager	Corporate Foundation Telecommunications	Non-profit
15	Didi	Communications Manager	Environmental NGO	Non-profit
16	Teodor	Founding Director	Health and wellbeing NGO	Non-profit
17	Alina	Community Development Manager	Minority Groups NGO	Non-profit
18	Ioana	Fundraising Manager	Global Environmental NGO	Non-profit
19	Silvia	Fundraising Manager	Health NGO	Non-profit
20	Cezara	Corporate Responsibility Director	Global Car Manufacturer	For-profit
21	Angela	National Fundraising Manager	Education NGO	Non-profit
22	Maria	Fundraising and Communications Manager	Community NGO	Non-profit
23	Adrian	Executive Director	Global Community NGO	Non-profit
24	Niculina	Executive Director	Community NGO	Non-profit
25	Carolina	Fundraising Manager	Global Community NGO	Non-profit
26	Luiza	Corporate Responsibility Manager	Bank	For-profit
27	Bianca	Fundraising Manager	Community NGO	Non-profit
28	Cristina	Community Relations Manager	Corporate Foundation Telecommunications	Non-profit
29	Elena	Executive Director	Community NGO	Non-profit
30	Gabriel	Programme Coordinator	Human Rights NGO	Non-profit
31	Cristian	Programme Director	Global Community NGO	Non-profit
32	Ramona	Executive Director	Community NGO	Non-profit
33	George	Director	Education and Community NGO	Non-profit
34	Alina	Programme Director	Education and Community NGO	Non-profit
35	Alan	National Director	International Human Rights NGO	Non-profit
36	Carla	Corporate Responsibility Manager	International Bank	For-profit
37	Mihaela	Director	Community NGO	Non-profit
38	Mary	Corporate Communications Manager	Global Technology Corporation	For-profit
39	Mihai	President	Health and Community NGO	Non-profit
40	Adina	Corporate Responsibility Manager	Insurance Company	For-profit
41	Mihnea	President	Community NGO	Non-profit
42	Alexandru	Executive Director	Corporate Foundation Health	Non-profit
43	Simona	Community Relations Manager	Corporate Foundation Retailer	Non-profit
44	Mariana	Fundraising Manager	Community NGO	Non-profit
45	Valentina	Corporate Communications Manager	International Construction Company	For-profit
46	Sandra	Executive Director	Community NGO	Non-profit
47	Daniela	Public Relations Manager	Global Construction Corporation	For-profit
48	Dorian	Director	Consulting Company	For-profit
49	Florin	Programme Coordinator	Minority Groups NGO	Non-profit
50	Sarah	President	Environmental NGO	Non-profit
51	John	Corporate Communications Manager	FMCG Corporation	For-profit
52	Laura	Corporate Relations Managers	Global Environmental NGO	Non-profit
53	Tony	Corporate Responsibility Manager	Cosmetics Corporation	For-profit

Participants were recruited through CSR networks, industry conferences and networking. Ethical procedures were followed in data collection and storage, and all interviews were recorded (with the permission of participants) and then transcribed. Two Romanian researchers conducted the interviews, but as not all the research team spoke Romanian, transcripts were independently translated into English for analysis purposes.

Interviews were open-ended, non-structured and non-directed, allowing us to better understand the everyday experiences of individuals as they “mobilize interpretive repertoires” (Gill & Larson, 2014, p. 528). Participants were asked about their careers, current roles and activities, including who they work with and what that work entails, and their experience of corporate responsibility outcomes. Participants were allowed to direct the focus of interviews in the ways that made most sense to them. The interviewers prompted them with questions such as: “Tell me about your organization”, “Tell me about your work”, “Tell me about who you work with” (i.e. co-workers, business partners, government administrators, or beneficiaries), “Tell me about CSR activities and their outcomes”.

In adopting something like Holstein and Gubrium’s (2005) interpretive practice approach, we then bracketed out existing normative and instrumental CSR definitions, to focus on practitioners lived experience. We then connected these accounts with broader societal discourses, recognizing that experience involves the use of language drawn from social meanings in sense-making (see Shutz, 1967, as a common reference point for phenomenology in business-related research). For Holstein and Gubrium (2005), we can connect individual discursive practice (meaning-making through language-based accounts) with broader discourse in practice or the social meanings from which experience must be understood. In doing so, we avoid the de-emphasis of structuring contexts (see Askegaard & Linnet, 2011). Our approach is to theorize the morality underlying CSR in Romania, free from pre-understandings of CSR, but sensitive to context.

Data interpretation was therefore iterative. Transcripts were read to establish global themes by comparing narratives identified in each interview across the dataset, then going back and forth between theorization and the empirical data. In this way, we aimed for a *merging of horizons* between researcher and participants and, ultimately, the reader, to allow them to understand what it is like to do CSR in Romania, the consequences of such undertakings, and how these experiences represent the broader cultural context. Key to our interpretation is the way in which participants expressed a desire to do good through CSR activity, but at the same time told us about the struggles they faced in advancing such projects, and of their instrumental engagements with CSR. As this ambivalence emerged, we sought a suitable sociological lens as enabling theory, and Bauman’s was selected was appropriate.

As the CSR community in Romania is small, and our data includes well-known practitioners and projects, to ensure anonymity we have used pseudonyms to represent practitioners and removed or modified identifying information such as age, the names of organizations and beneficiaries. We are also aware that participants may come across in a negative light in our interpretation (for example, as cynical). We should therefore make clear that criticism is not directed towards our participants, but rather at the structures within which they practice CSR. Our overall impression was that participants wanted good to happen in Romania, and their behaviours were expressed as ‘normal’, ‘common’, or ‘how things are in Romania’. We are therefore suggesting that this is how Romanian CSR is understood to happen, rather than commenting specifically on our participants.

We avoid counting, consistent with Hannah and Lautsch’s (2011) view that doing so is inconsistent with the goals of theory-advancing qualitative research, for example, because it backgrounds the interpretation of complex experiences in favour of common ground within narratives related to established theory, so limiting researchers’ ability to generate insights from unexpected findings. We further acknowledge Driver’s (2017) argument that interpretivist studies aim for plausible interpretations of a phenomenon rather than causation or correlation, and Rynes and Gephard’s (2004, p. 455) view that qualitative research “starts from and returns to words, talk, and texts as meaningful representations of concepts”. Therefore we do not offer a measurement of variables relating to CSR, but rather we provide plausible explanations of the experiences of our participants and related structures of meaning in order to theorize CSR in Romania.

## Findings

We organize the findings into four sections: the Romanian context, organizational processes, individual practice, and the consequences for Romanian CSR.

### Experiencing Romanian Culture

Discussions with participants acknowledged current news stories, recent corporate and EU developments (including in CSR), and Romanian history and culture. For participants, a scandal over Roşia Montană (a natural area connected to recent political corruption scandals around mining interests) and the fire at the Colectiv nightclub in Bucharest (that confirmed the belief that officials take bribes from businesses to avoid compliance with legislation), were recent reminders of a normalized culture of corruption.

However, the introduction of CSR through multinationals and EU initiatives was presented as allowing for job opportunities and personal success (rather than a solution to the broader problems of Romanian society). Both NGO and corporate participants noted personal achievements, along with their acknowledgement of significant limitations in making meaningful change happen. Frequently, they would resort to statements like “this is how it is in Romania”, or “this is just how things work”, consistent with what Bauman and Donskis (2016) report as a feeling that “there is no alternative”, and with Borţun’s (2011) belief that Romanians too often accept a lack of progress as a characteristic of Romania.

Again consistent with Borţun’s (2011) observations, the focus in accounting for the cultural context was communism. Participants described the communist era as a time when the state would organize their lives, but where there was never enough of anything. They also recognized that there was a break, or schism following the 1989 revolution that hailed a new entrepreneurialism, especially in the young, and that later entry into the EU (in 2007) invited further opportunity to benefit from markets and investment. For most though, these promises are unrealized. Dumitru, a senior figure at an environmental NGO, explained a change in sentiment following communism, noting the chaotic, competitive and insecure situation that resulted:

[we are in] recovery, the convalescence of a nation after 45 years of serious trauma... We thought that capitalism would be all settled in 10 years for us, and done! In ten years, we’ll be like in the West. And we had the disappointment to see it’s not so. Capitalism is actually much more difficult than communism, because capitalism is so competitive, it’s so uncertain, it introduces an element of insecurity… That’s the problem with the average Romanian. He got out of communism, good, it was very nasty, what happened was wrong. We put everything aside and we went into capitalism. We don’t know how to implement all the capitalist components correctly though; it doesn’t work properly.

For Romanians who were used to state organization of much of their life, albeit with shortages in basic goods and services (Al-Khatib et al., 2004), capitalism is experienced as insecure and uncertain, yet with the promise of personal gain. Dumitru’s reference to an inability to implement capitalism, however, is also consistent with Borţun’s (2011) view of Romania as lacking the necessary social, political and legal ‘values’ necessary for a modern capitalist society. Mugur, who works at a human rights NGO, explains the implications of this:

We all had the opportunity to access more generous funds, more money. And I saw a change in attitude. All of a sudden, people were very business-oriented… I mean it’s ‘business-oriented’ when you have no scruples [literally, ‘step over corpses’] … all of a sudden you’re only interested in discussing the budget, working as little as possible and being allocated as much money as possible.

Participants felt they were playing catch-up with Western Europe, characterized by what Bauman and Donskis (2013, p. 44) describe as “change without any chance left to slow down and think for a while”. Ethics are affected as attitudes emphasize a pre-modern individualism more than socialist collective aims, and therefore individual competitiveness over thoughts about social progress. Following EU investment, an ‘every man for himself’ mentality remained, characterized by a desire for personal gain, and reaffirming Borţun’s (2011) concerns about an older, medieval view of individualism that is parochial in its self-interest. This social context influences both the focus and purpose of CSR activity following EU membership.

### Distancing and Externalization in Romanian CSR

Ethical business practice was seldom directly referred to and was often conflated with legislation (“we comply with the law”), consistent with Crotty’s (2016) observations from Russia. More often, CSR managers revealed how businesses are discursively distanced from morality in favour of talk of business performance. Georgina, who works at an NGO that supports public health, explains corporate motives (as she understands them):

Everything the company does, is meant to make or support that business. And that means that you don’t do CSR just like that, out of the goodness of your heart. There’s a very low interest for investing in things that are not popular. We like concrete things because it’s easy to report about them, we write the CSR report at the end of the year, and then say ‘goodbye!’

Consistent with Borţun’s (2015) concerns, CSR reports are described as serving an organization’s promotional requirements and so avoid difficult or contentious social issues. This also means that unlike Frederiksen’s (2010) suggestion of a preference for projects that are close to the expertise of an organization, projects may specifically avoid such proximity. For example, a participant who works at a corporation whose products have been linked with health problems reported that they specifically avoid NGOs that deal with health. Corporate participants told stories of aligning only positive values with the promotional opportunities afforded by specific NGOs, and of avoiding others as “not right for us”, even where they may serve a more pressing societal need (see Smith & Higgins, 2000). CSR is understood as a marketing activity rather than an ethical commitment, and anything unrelated to promotional aims was placed outside of the concern of CSR practitioners. In addition, the business requirement for frequent reporting, meant that long-term projects were deemed unsuitable and therefore also distanced from consideration. In these contexts, CSR activity is about managing a short-term process relating to the generation of opportunities for positive representations of the organization.

Where ethics were specifically discussed, it was in relation to managing *employees’ behaviour* in the interests of the organization. Diana, a manager responsible for CSR at a subsidiary of a global cosmetics company, explains that employees undertake ethics training that deals with *internal* fraud. Ethical practice is primarily about protecting the company from employees’ misbehaviour. Through her recollection of a special ethics event, she then explains how ethical practice is directed towards individual employees’ behaviour, even when it is part of CSR activity. The event is designed to get employees to think about how they can help the community:

On the [event day], we wanted [employees] to be as relaxed and as focused on ethics as possible, and not focusing on work. People from accounts declared: ‘On [event day], we’ll dedicate all our energy to helping the community instead of calculating results. What are you going to do?’... The boys from sales: ‘We’ll support new lives instead of new selling propositions,’ all with call for action: ‘What are YOU going to do? What are YOU going to do?’…. Then HR: ‘Instead of developing new talent we develop solidarity. What are YOU going to do?’… And us, in CSR: ‘Instead of spreading information we’ll be spreading hope. What are YOU going to do?’

In this story, focussing on ethics as part of CSR is *not* focussing on work. Diana understands CSR as opposed to what the organization usually does. The event represents something like a contemporary festival of inversion (see Featherstone, 2007), confirming the usual absence of ethical concerns as normal and distant from the routine of work, and placing ethics outside the organization, on just this day. The calls are also individualized, with responsibility put in the hands of employees: “what are YOU going to do”, and not what “we” or “the organization” should do. Again, the emphasis is not on establishing ethical business practice, but on how individual employees can make the organization look good while organizational activities remain outside moral consideration.

We also heard how responsibility for ethical problems could be passed onto external stakeholders, further externalizing responsibility. For example, John works in communication at a FMCG manufacturer. He tells us about a project on water use where the corporation is explicitly distanced from such concern and, instead, families are encouraged to reduce their consumption:

The impact is not in our factories, but with the consumers, in how much water *they* use. They use a lot of water in the shower… how much water they use when they wash the dishes… so that’s why we focus on changing the consumer, and not the factories, we’re absolutely green in the factories … so we have to work on the consumer.

In the most recent interview (2016), John also tells us that the recycling company his organization worked with were fined for failing to recycle the organization’s waste effectively. Although this is presented as beyond the organization’s responsibility, the failure resulted in his organization not meeting its EU target (with associated access to grants). The story unfolds with his company (and others) lobbying government to change the law to make compliance easier for them, and to further pass responsibility onto contractors, with the government as enforcer of the contractor’s obligations rather than the company. This not only illustrates distancing, but also the reduction of ethical concerns to bureaucratic measures. Discussion is not about achieving sustainability, but rather about the structures of accountability and technical enforcement that make compliance easier. Indeed, there is much discussion of the bureaucracy involved in CSR activity, including frequent reference to how projects were funded and reported. Sabina talks about the funding criteria for NGOs that apply to the corporate foundation she works for:

All our partners have to demonstrate that the project that gets funding can report monthly; both financially and creatively. All projects are carefully monitored to make sure that money is used properly.

Corporate morality is reduced to the bureaucracy of monitoring external partners. Doing good exists in the monitoring process rather than the actions of the organization. Later, we learn that ‘properly’ means the generation of activities with promotional value.

By fragmenting roles and projects so that their focus is narrow and bureaucratized, distancing is achieved and responsibilities externalized. This is consistent with Jensen’s (2010, 2014) analysis of adiaphoric organizations. CSR is organized so that it distances organizations from any long-term commitment to causes and from any contentious or unpopular issues. CSR practice places moral action outside the everyday work an organization does, passing the ethical buck (Bauman & Donskis, 2013) onto stakeholders. As we shall see though, these same processes also create insecurity and competitiveness in CSR, and may invite corruption.

### The Morality of Romanian CSR

It is within this context that we observed a lack of reference to societal progress or even to organizational missions (ironic, given the apparent instrumentality), in favour of individual narratives of success. Although participants may mention the alignment of CSR with the brand, they emphasize personal reward, achievement and recognition as the important parts of their CSR role. For example, when asked about the outcomes of a project, Beatrice, who works in the finance sector, starts by talking proudly about winning an award:

I coordinated the reporting activity. I kept in touch with the suppliers, I answered questions, I saw that on the indicators we had chosen were reported as completely as possible… [the corporation] were the first and more importantly the only ones who did this, and we recently received an award for that.

Effective CSR work is understood as that which enhances status and recognition in the industry, which makes the practitioner appear good to peers. Yet, this also means that there is no commitment to any role. Participants presented working biographies that involved various moves between organizations and sectors, and in and out of CSR. In an interview in 2014, Tony expresses a desire to move from one challenge to another for personal and professional development. In a later interview in 2016, after she moved from CSR to a PR role, she confirms:

I started as a PR assistant. After nine months I got to coordinate the CSR activity. This job brought me great satisfaction… I was the only person in the company responsible for this and gained visibility and exposure. But I’m not the kind of person who gets attached to one field of activity, rather I’m interested in my professional development. So, at the time, it was the natural step to move away from coordinating CSR activity and get back into PR and advertising.

Tony is not ‘for’ any cause, but rather seeks professional progression and self-exposure. She tells us that her interest in CSR is about being seen by and seeing the right people, or “those who matter”—as Bauman and Donskis (2013) describe—people with influence and who may contribute to professional development. Mugur tells us a similar story, highlighting his extensive business contacts then explaining how he sacrifices his private life to succeed:

I am so busy that I don’t really get to see my friends, I avoid phone calls and invitations as much as possible because I feel like I don’t have time for anything. I don’t take much time off for vacations. As an individual, I’ll be slightly arrogant here, but I succeeded in making a certain image for myself in this organization… [others in the organization are] more senior, but under me in [CSR] projects.

Our participants exhibit the characteristics of labour that Bauman and Donskis (2016) have identified, favouring connections that improve their careers. Their focus is directed towards those more senior, and on meeting organizational criteria for personal success. We see a morality in CSR where self-interest defines what is good (Roberts, 2003).

The process is competitive, but also insecure, especially for NGOs workers who express a need to make the strongest case of suffering and to provide the best metrics of improvement, along with engaging stories, to achieve corporate funding. George works on community projects for an NGO and explains the relationship between NGOs and corporations as he sees it:

I’m still familiarizing myself with what companies want from NGOs, and I note that mostly they want PR. I had questions like ‘do you do CSR with media relations attached to it?’... Another thing that I stumble upon quite often is that all the money that you get from companies need to go on activities and beneficiaries... it is hard to convince companies that the money they give you needs to go on people’s salary [in the NGO].

Corporations ask NGOs (external organizations) to ‘do their CSR’. George further explains how NGO staff must be funded from other sources, to maximize the claimed results for the corporate sponsor, while externalizing project costs (although the lack of trust may also be a result of experiences or perceptions of corruption, see below). Yet he then explains that almost all funding for NGOs come from corporate sponsors. This reveals an instrumental relationship between corporation and NGO. An implication is that NGOs financing (and so also the projects they undertake) is precarious and may exist only for as long as project funding lasts. Florin, who works at the Romanian branch of an international NGO that supports minority groups confirms: “after the money from the project dries up, what is the value of such project? Most people produced a plan, but it was all a big fantasy”*.* The hazardous nature of funding, and the desire for career progression, without stability or certainty, means that individuals go wherever they can achieve their personal or professional goals (see Bauman & Donskis, 2013). This supersedes long-term responsibility to either causes or organizations. Participants gave biographical accounts of moving between jobs, working on multiple projects at the same time, and moving between corporations and NGOs. CSR projects are experienced as opportunities for self-development, personal satisfaction, or pay. We should make clear that we are not suggesting cynicism in participants, rather this is how they account for what is normal practice in their view. Florin explains the implication of such projectization and individualism on NGOs:

People either work for a paycheck for a time, or they work for as long as the project is running and then they leave. That’s how it was with us, or at least for me, a string of different projects.

Participants are ‘with’ but not ‘for’ a cause or organization. CSR in Romania is presented as a series of small-scale, temporary and episodic projects, dropped when money or publicity expire. In this context, individuals and causes are encouraged to compete, with timeframes compressed through a bureaucratized morality that reduces doing good to monthly returns and measures of promotional value. The result for our practitioners is ambivalence about their reports of self-interest and the experience of wanting to do good.

### The Consequences of Romanian CSR

Participants recognize a moral distancing from beneficiaries and a focus on how both they, and the organizations they work for, appear to others. For example, Laura who works in corporate relations at an international environmental NGO, explains a CSR manager’s role as “laying a smokescreen” of small projects for external audiences to engage with, specifically to avoid scrutiny of other organizational activity. CSR activities become deliberately situated outside the organizational boundaries and distanced from the organizational strategies that they are meant to be embedded in (not unlike the decoupling observed by Jamali et al., 2017). Dumitru states:

Another company trick is this: they have divided a much smaller budget to much smaller projects. So you read on their website: ‘we had 20 CSR projects’ all with fancy titles, but if you look at the results and the effect of those, if you go bottom up, you’ll have the surprise to see that they actually spent 5–10,000 euros on a project where half of that money was just [corporate] expenses.

Ramona, an executive director at a community NGO, provides a similar account, again recognizing the implications: “the social impact is reduced… change is not seen in the long term. And in general, small projects tend to produce short-term effects”. There is another significant consequence of such short-term focus. Didi, a communications manager at an environmental NGO, explains:

When you go to a company, you are in competition with other NGOs that might have better projects than us. In the end, the project with the best costs will win. So, there is this competition, just as there is competition between companies.

Maria, from the local office of a prestigious community NGO, confirms: “there is a tacit confrontation between NGOs, because they fight for the same resources.” Maria expresses a marketization of suffering in Romania that Borţun (2015) has also raised concerns about. NGOs become like businesses (see Maier et al., 2016), not because of the efficiency of business processes, but because they must satisfy the requirements of corporate sponsors, and these relate to demonstrating low costs and value for money in terms of promotional potential. This means focussing on funding, and viewing other causes as competitors. In turn, corporations consider projects on the basis of their immediate return on investment, encouraging and benefiting from competitive suffering where everyone’s attention is on the next engaging story about doing good. This creates a façade of progress as a series of good news stories that mask an absence of meaningful long-term change.

Fraud and corruption also emerge from such a context. Participants conveyed that this was common, resulting from limited legitimate opportunity, the possibility of personal gain, a lack of legal sanctions, and most significantly, a detachment from those affected. We recognize the vulnerability of participants here and note that they did not directly confess to crime and were not encouraged to do so. Indeed, participants brought up the topic spontaneously, speaking in general terms more often than specific cases. Florin provides a story about a visit from a government inspector who suspects that those working on CSR are fraudulently claiming money from several projects:

[the auditor] says: ‘Look at the salary this guy has in the project! Let us go and check him out!’…Their comments made you ashamed, something like: ‘Come on, you people, who work in projects like these, we know you got your houses through that program…’ They ask: ‘Why do you have two contracts for two projects? And why do you work in three projects? What are you doing with all that money?’… The interesting thing is that they [auditors] also try to jump ship! And all of a sudden, it’s: ‘You are earning too much, brother, let’s see why you are earning so much’

The desire to do good is experienced alongside a felt need for personal financial reward in the context of precarious funding, and this might be why corporations are reluctant to pay a contribution to NGO partners’ salaries. Florin’s story includes suggestions that there is a doubling-up of reported benefits. There is an absence of responsibility to potential beneficiaries, and instead the focus is on bureaucratized monitoring. As documents are distanced from the people they represent, nobody ‘suffers’ in their fabrication. Only when the auditor directly questions activity is there a moral emotion of shame. Florin returns to the topic in a later interview (2016), telling us more about his understanding of the limitations in the Romanian legal system:

People wrote a grant, then took the money and said ‘goodbye!’ Someone would write up the paperwork, then embezzle money… Now, I notice that in some cases, the authorities do something about this, they investigate, but in a superficial way, they will never catch all those who took money.

Corruption results from a moral distance, projectization and a bureaucratization of adiaphoric organizations (Jensen, 2010, 2014). In creating morally insensitive, insecure employment that distances employees from potential beneficiaries, a numbness of spontaneous moral impulse becomes apparent. Sarah, who has a senior position at an environmental NGO, adds CSR departments to the list of potentially corrupt entities:

Companies don’t have a conscience per se. They don’t! They’re not regulated, many work with bribes, the bribes get paid precisely from the CSR and communications budgets. Most companies use bribes like crazy.

Florin feels Romanian culture perpetuates corruption in organizations and this leads to systems of monitoring and governance. Hence Diana’s stories of internal ethics training (above). But the external consequences are more severe. Corruption and inefficiencies result in little social progress despite the apparent commitment to CSR activity and indeed we might recognize that as long as corporations get their desired reports, they may have little interest in actual social benefits, in the same way as John is content that his organization can sign-off on its recycling commitments, regardless of whether or not the contractor actually deals with waste responsibly.

The myth of CSR (Devinney, 2009) unravels in the tension between individual self-interest in an insecure and competitive work environment, and a greater social good. Even those who are trying to improve society may succumb to corruption and individuals feel unable to grasp a way of changing these aspects of culture for the better. Yet this does not mean that practitioners are entirely stripped of moral impulse. Within the stories of personal success, of awards, of project reporting and of corruption, there are examples of beneficiaries being helped. Iulia, a CSR manager at a large multinational gives one account:

The director read a newspaper headline about an old woman living on the streets, without relatives, without any food, without shelter. So deplorable! And then I went to the director of community relations and said, ‘Look, please do something nice for her.’ And then I appealed to the district mayor [...] with the support of City Hall we basically selected a number of old people who were at risk ... [These] beneficiaries got a hot meal, delivered directly to them each day.

A news report results in a series of interpersonal exchanges involving appeals to compassion that are taken up and acted on to produce an intervention. Florin tells us about a person with limited education who came to see him at the NGO he works for:

Another case was an uneducated guy. He had such enthusiasm. He came one winter to our office; it was a very harsh winter… He was like: ‘I want a job’. I racked my brain. Where in God’s name could I take him? Eventually, I found a cleaning firm. We sorted something out.

Niculina, an executive director at a community NGO, explains how she managed to provide food and educational opportunities for disadvantaged children with the help of a corporate sponsor. Tony tells of her corporate CSR project that helps train women in a shelter and of one who subsequently got a job. John reports on eight families who saved money as a result of his consumer education programme. These stories provide evidence that the moral impulse is not extinguished in the corporate and social context that we have described. However, in each case, the story focuses on just a handful of beneficiaries. They are contingent, almost accidental to the corporate purpose in undertaking CSR, and instead result from human encounters and interventions.

## Discussion

**Fig. 1:**
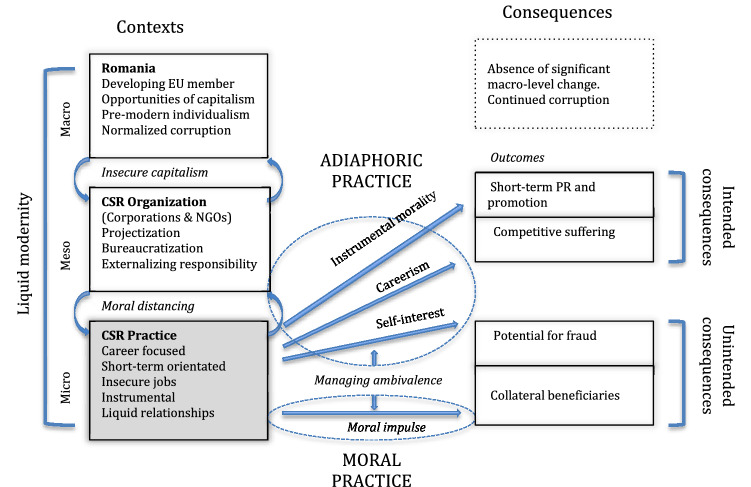
Liquid CSR in Romania

Participants articulated Romanian CSR as characterized by both the opportunities and insecurities of post-communist capitalism, with a culture where corruption is normalized. However, the individualized view of CSR activity that is accepted as ‘just how things are in Romania’, also resonates with Borţun’s (2011) observations of limited structures of citizenship, at both political and corporate level. At the organizational level, this is manifest as fragmented roles, projectization, bureaucratization, and moral distancing that results in ethics being externalized, ensuring that corporate activity is *not* the subject of moral evaluation. Rather, attention is diverted to the promotion of CSR projects as *external* evidence of the virtuous corporation (Köllen, 2016). Romanian CSR is not just market driven (Crotty, 2016) and explicit (Matten and Moon, 2006), but is adiaphoric in that it places the organizational activities outside moral consideration. At the individual level, there is a short-term orientation (including job insecurity and careerism), instrumental approaches, and ‘pure’ relationships. CSR also exploits what Bauman and Donskis (2013) describe as *competitive victimhood* in response to corporate promotional KPIs. This results in both the unintended consequences of corruption, but also ‘unintended’ beneficiaries. The individual moral impulse is not completely suppressed, but given the lack of corporate moral intent, we might theorize the few who are helped as mere *collateral beneficiaries* of adiaphoric practice (see Fig. 1). We now consider these ideas in more detail.

### Moral Distancing and Adiaphora

The opportunities for individual wealth and achievement promised by capitalism, combined with ineffective Romanian governance processes, meant that CSR managers focus on career enhancement and NGO managers on securing funding, rather than the societal change that seems to have long evaded Romania (see Borţun, 2011). One result is the conditions for adiaphora in business practice described by Bauman and Donskis (2013), and what Jensen (2010, 2014) calls the adiaphoric organization, characterized fragmented roles, bureaucratization, projectization and moral distancing. Participants talk about documenting outcomes that meet organizational performance criteria, but that are narrowly defined within a specific role and timeframe and even separate from what might actually happen. The result is that CSR amounts to small, short-term projects, with little impact, negotiated through reports and spreadsheets. Although consistent with Van de Ven (2008) observation of *consequentialism* driving CSR towards arbitrary selection of causes based on communication strategy, a difference is an absence of obvious ‘win–win’ outcomes. Borţun (2011) notes a tendency in Romania to create ‘façades’ rather than substantial change, and we can see this in CSR activity that presents corporations and NGOs as doing good through external images of social progress, while meaningful and long-term engagement is absent. Such processes also distance practitioners from the possible beneficiaries of their activity, allowing them to accept and reproduce de-moralizing organizational structures. CSR practice exhibits a narrow view of who matters, and an absence of care in both internal and external relationships. This is consistent with the construction of adiaphora in organizations (Bauman & Donskis, 2013; Jensen, 2010, 2014), and Jamali et al.’s (2017) ‘decoupling’ strategies.

This also challenges existing (largely Western) CSR-related conceptualizations such as strategic corporate philanthropy (Liket & Maas, 2016), corporate citizenship (Matten & Crane, 2005), or shared value (Porter & Kramer, 2011), even as they confirm Bauman’s view of an individualized society. Normative claims about the responsibilities organizations should take on require a moral structure that participants rarely reflect on, and that is outside their daily concerns. This is partially aligned with Pedersen’s (2010) conclusions that managers’ perceptions of responsibilities towards society differ from the mainstream models in CSR and business ethics literature. Instrumental CSR approaches relating to competitive advantage (Porter & Kramer, 2011), increased financial performance (Luo & Bhattacharya, 2006), or enhanced brand awareness (Bhattacharya et al., 2009) might at first resonate more, yet we also note an absence of clear alignment between corporate strategy and purpose in the short-term pressures of reporting, and the more worrying suggestions of corruption, heightened by Romania’s previous limited success at embedding modernist ideas in institutions.

### CSR Practice and the Individual Moral Impulse

The cultural patterning of CSR in Romania is therefore also unlike that based on *Confucianism* in China (Wang & Juslin, 2009; Yin & Zhang, 2012) or *Kyosei* in Japan (Boardman & Kato, 2003), and still dissimilar from the *coercive* CSR that results from the Russian context. Bauman’s ethics of responsibility better helps us understand Romanian CSR as it reveals that rather than taking responsibility for others, discursive practice is to avoid it. Ethics is not the organization’s daily concern, even as the organization lays public claim to being virtuous through reports of external activity. The people we spoke to attempt to make both themselves and the organization look good to others (see also Roberts’, 2003, narcissistic morality), while avoiding scrutiny of what the organization actually does. Criteria for evaluation explicitly emphasize promotional and career prospects, and so NGOs are invited to pitch the ‘suffering’ they want to address to CSR managers on this basis.

The organizing logic is financial gain (often simply enough to get by when jobs are precarious) and, related to this, the desire to secure recognition. CSR practitioners identify the causes that are most likely to achieve visibility and funding. Without an apparent belief that a better Romania may be built, participants are, as Bauman (2001) describes, ‘alone, and in it for themselves’, at the sharp end of the consequences of individualized societies, exhibiting little compassion (Köllen, 2016). The fear of unimportance, of leaving no trace (Bauman & Donskis, 2013) frames CSR practice. Practitioners take pride in their individual achievements and status, but no cause, no company, no beneficiary is permanent. Even auditors may ‘jump ship’. This means being ‘with’, but not ‘for’ organization, cause or beneficiaries.

Yet careerism and self-interest do not entirely suppress the moral impulse. Participants also talk of their *desire to do good*, of their frustration at not always doing so, and of their witnessing of suffering that may be alleviated. The result is ambivalence about the conditions under which they feel they must operate. Participants move between their accounts of self-interest, and their instincts for moral practice, highlighting to us the specific beneficiaries they have helped.

### Liquid CSR in Romania

Romanian CSR is therefore promotional, bureaucratic, short-term, subject to arbitrary change, and outside of moral concerns, or what we might refer to as ‘liquid’. It involves ‘passing the ethical buck’ (Bauman & Donskis, 2013) to employees, consumers, contractors or government. For example, we heard of employees encouraged to consider what they might do in the name of the organization (not what the organization might do for them or for society), of water waste as a consumer issue (not an organizational issue), and of moves to get the government to enforce contractor recycling obligations (instead of assuming organizational responsibility for sustainability), and of reports of CSR achievements being more important than evidence of actual results. CSR work deflects attention away from corporate activity as an act of misdirection. For this to work, the conditions of liquid modernity are required as individual actors remain focussed on the self, so that there is no time for reflection.

In Romanian CSR, suffering is *marketized*—to be consumed as brand value—as managers consider which cause best speaks to their stakeholders at the lowest cost, or what Bauman and Donskis (2013) call competitive victimhood. NGOs compete to meet requirements, recognizing the need for quick and impressive results and viewing their fellow NGOs as competitors. CSR focuses on constructing marketable causes that are most attractive in terms of brand values and publicity, implemented through small NGOs, each potentially as transient as their grant, and competing with each other.

An unintended consequence is fraud and corruption as ways to secure financial gain in an environment where employment is precarious, and where NGOs materialize in response to funding opportunity, and then evaporate when funding expires. We heard stories about the fabrication of documents to ensure bureaucratic criteria were met. As Jensen (2014) notes, this is not a case of a few individuals that might be better trained or managed. Rather, these practices are normalized. In the words of participants, ‘this is how things are in Romania’.

Yet our participants *can* point to the few people they have helped, the *collateral beneficiaries* of adiaphoric practice. Bauman (2011) describes ‘failed consumers’—those who cannot reap the rewards of globalized markets—as the collateral damage of capitalism, because capitalism distanced itself from their plight, but meant them no harm. In Romanian CSR, we see individuals benefiting from the action of the same corporations who now find it convenient to turn to them for their own instrumental morality, but are not ‘for’ them, not committed to their welfare, or long-term prospects, and so actually take no responsibility for them.

CSR makes claims about the ability of the corporation to be a good citizen, just as social commentary notes a reduction of the citizen to a consumer (Bauman, 2013, 2016). This is compounded in Romania where modernism itself may be understood to have ‘failed’ (Borţun, 2011). Romania has not managed to achieve the level of governance that Bauman notes has eroded elsewhere, but has moved from one type of individualism to another, represented in the practices of CSR that we have described. CSR cannot therefore replace grand claims to (social) progress promised by modernism while maintaining an adiaphoric approach to corporate responsibility, it can only dilute them. In Romania, they were never fully formed to begin with and, under liquid modernism, we see the instrumental version of CSR that our participants describe.

## Conclusion and Future Research

Whereas previous research seeks to consider either the strategic value of CSR, or the morality that best underpins it, further deriving critique from both positions, we suggest that CSR practice may sometimes evade morality, failing to ‘do well by doing good’. Our interpretation of *liquid CSR* is therefore in contrast to both normative/ethical and instrumental/strategic CSR claims, both of which might be seen as perpetuating a myth of modernism: the possibility of social progress through corporate citizenly activity. Although instrumental CSR theory offers a closer match to the ethics present throughout our interviews, this fails to account for the individualized, self-interested, small-scale approach to CSR projects.

Whereas Bauman (2011) laments the collateral causalities of our consumer culture, here we see merely *collateral beneficiaries*, resulting from the contingency of short-term promotional activity rather than from corporate purpose, or responsibility. CSR is episodic and unstable, and ‘doing good’ is little more than where morality drips though the gaps in the adiaphoric organization. When responsibilities are blurred as they are here, the conditions of moral blindness are perpetuated. In a culture where corruption is normalized (Borţun, 2011), issues of ethical business behaviour may be dismissed as distant from the everyday lives of our participants. Bystander apathy becomes inevitable and corporate purpose remains outside moral consideration by ensuring that discussions of both the good they do and harm done in society are externalized. Although we recognize that such a claim might seem glib, we share Jensen’s (2014) hope that these mechanisms can be reversed. For us, this requires the creativity and persistence by the Romanian people to create the necessary broader understanding of democratic structures, or citizenly responsibilities, and not just more CSR projects that perpetuate a façade of progress through a network of precarious NGOs.

Although we do not doubt that these stories represent the experiences of our participants (phenomenology takes, as its starting point, the authenticity of reports of lived experience), we cannot claim universal or even general claims about CSR through them. *Liquid CSR* is therefore just one plausible theorisation of Romanian practice. It is disappointing to see both investment and human energy being diverted from the needs of the people in Romania, but what is revealed here might also be true elsewhere, even if less obvious and/or moderated by the relevant local cultures. To understand this, scholars might therefore shift their focus towards the moral conditions under which CSR is practiced, and to the methodologies that may best capture this. Our theorizing is ‘local’ to the experiences of those in responsible careers, but it may be transferable through the ‘phenomenological nod’ (Manen, 2016) of recognition to other contexts where there may be a tension between individual moral impulse and an instrumental morality. It may therefore inform other studies of interpretive practice in new cultural contexts. Further, we do not capture all voices involved. The experience of government officials, shareholders, consumers or the beneficiaries themselves may provide additional perspectives, as might organizational documents or broader data on CSR and macrosystems.
